# Editorial: Advances in two-dimensional materials for electrochemical energy conversion and storage

**DOI:** 10.3389/fchem.2025.1724936

**Published:** 2025-12-08

**Authors:** Chenhui Yang, Yi Tang

**Affiliations:** 1 School of Chemistry and Chemical Engineering, Northwestern Polytechnical University, Xi’an, Shaanxi, China; 2 College of Materials Science and Engineering, Xi’an University of Science and Technology, Xi’an, Shaanxi, China

**Keywords:** two-dimensional materials, synthesis process, modification strategy, electrochemical energy conversion and storage, electrochemical techniques

Due to the demand for fast-charging consumer electronics and electric vehicles, high power density is essential for advanced electrochemical energy storage devices. As an electrochemical energy storage device characterized by both excellent capacity and power performance, supercapacitors hold significant importance for technological development. Recent studies in materials science and electrochemistry have convergently focused on the intrinsic connection between the internal structure of materials and their macroscopic properties. Achieving an optimal balance among various performance metrics of materials poses a central challenge for electrochemical energy storage and sensing technologies. A series of investigations demonstrate that the key to addressing this challenge lies in understanding and harnessing the structure-property relationship of materials at the microscopic scale. From vacancy engineering and interface design to AI-aided prediction and composite structure modulation, scientists are exploring multiple pathways to elucidate how structure governs performance.

The first article by Tang et al. is aimed on the influence of vacancies on the electrochemical properties of 2D MXenes and the manifested structure-property relationships. Research indicates that MXenes contain homogeneous metal vacancies resulting from over-etching of the M-layer during the etching process, heterogeneous metal vacancies with ordered arrangements achieved through selective etching, as well as carbon vacancies originating from defects in the precursor. These vacancies facilitate ion migration within the material, thereby enhancing electrochemical performance. Strategic vacancy engineering can ultimately break through the intrinsic capacitance limitations of materials.

The second article by He et al. is focused on electrochemical sensing devices and the study of their performance. It presents a highly sensitive, disposable electrochemical aptasensor utilizing amino-functionalized vertically-ordered mesoporous silica films and an electrochemically polarized screen-printed carbon electrode for the rapid and sensitive detection of carcinoembryonic antigen. The research further elucidates the influence of structure-property relationships on the electrochemical performance of the materials.

The third article by Khan et al. discusses about the application of artificial intelligence in drug design, reviewing its key application areas in drug discovery—including target identification, virtual screening, *de novo* drug design, toxicity prediction, and retrosynthesis prediction—while also discussing current challenges and future directions. It highlights that structure-activity relationship, as the foundational logic of drug design, can also be applied to materials research when integrated with artificial intelligence and big data.

The fourth article by Asghar et al. advancements in the application of metal oxides and their composite materials as electrode components for safe and sustainable supercapacitors. It summarizes the structural characteristics and electrochemical performance of commonly used supercapacitor electrodes, demonstrating the correlation between material properties and architecture. Furthermore, the authors emphasize that the ion transport mechanisms—particularly the complex interfacial ion transport processes within composite materials—still require further in-depth investigation.

The fifth article by Adak et al. focuses on exploring the use of epoxy resin in developing multifunctional solid polymer electrolytes, aiming to simultaneously achieve high mechanical strength and excellent ionic conductivity for applications in structural batteries and structural supercapacitors. The study highlights that balancing robust mechanical integrity with high ionic conductivity remains the foremost scientific challenge in solid-state electrolyte design. To address this, understanding ion transport mechanisms—particularly the complex interfacial ion transport processes at filler/polymer, polymer/polymer, and electrolyte/electrode interfaces within composite electrolytes—is of paramount significance.

In summary, although these five studies focus on different material systems and application areas, they collectively reveal a core paradigm: a deterministic correlation exists between the intrinsic structure of materials and their macroscopic electrochemical properties. Whether through vacancy engineering, interface design, or prediction and optimization aided by artificial intelligence, a profound understanding and precise regulation of the “structure-property relationship” is key to achieving performance breakthroughs. Simultaneously, these studies unanimously emphasize that delving deeper into ion transport mechanisms—particularly the complex interfacial processes within composite materials—will be of paramount importance for overcoming current technical bottlenecks and advancing the development of next-generation high-performance electrochemical devices. Future research will continue to follow the philosophy of progressing from structural design to performance realization, paving the way for more efficient material design and development.

